# Fabrication and Application of SERS-Active Cellulose Fibers Regenerated from Waste Resource

**DOI:** 10.3390/polym13132142

**Published:** 2021-06-29

**Authors:** Shengjun Wang, Jiaqi Guo, Yibo Ma, Alan X. Wang, Xianming Kong, Qian Yu

**Affiliations:** 1 School of Petrochemical Engineering, Liaoning Petrochemical University, Fushun 113001, China; wsj203203@sina.com; 2 Jiangsu Co-Innovation Center for Efficient Processing and Utilization of Forest Resources and Joint International Research Lab of Lignocellulosic Functional Materials, Nanjing Forestry University, Nanjing 210037, China; jiaqi.guo@njfu.edu.cn; 3 Department of Bioproducts and Biosystems, School of Chemical Engineering, Aalto University, FI-00076 Aalto, Finland; yibo.ma@aalto.fi; 4 School of Electrical Engineering and Computer Science, Oregon State University, Corvallis, OR 97331, USA; alan.wang@oregonstate.edu

**Keywords:** regenerated cellulose fiber, Au NP, controllably assembled, SERS, dimetridazole

## Abstract

The flexible SERS substrate were prepared base on regenerated cellulose fibers, in which the Au nanoparticles were controllably assembled on fiber through electrostatic interaction. The cellulose fiber was regenerated from waste paper through the dry-jet wet spinning method, an eco-friendly and convenient approach by using ionic liquid. The Au NPs could be controllably distributed on the surface of fiber by adjusting the conditions during the process of assembling. Finite-difference time-domain theoretical simulations verified the intense local electromagnetic fields of plasmonic composites. The flexible SERS fibers show excellent SERS sensitivity and adsorption capability. A typical Raman probe molecule, 4-Mercaptobenzoicacid (4-MBA), was used to verify the SERS cellulose fibers, the sensitivity could achieve to 10^−9^ M. The flexible SERS fibers were successfully used for identifying dimetridazole (DMZ) from aqueous solution. Furthermore, the flexible SERS fibers were used for detecting DMZ from the surface of fish by simply swabbing process. It is clear that the fabricated plasmonic composite can be applied for the identifying toxins and chemicals.

## 1. Introduction

Cellulose is one of the most abundant biopolymers derived from biomass, which has been widely applied for synthesizing functional materials such as drug delivery, optical sensors, lithium-ion battery, textile, and biomedical engineering [1,2,3,4,5,6]. Cellulose is a cost-effective, eco-friendly, and biodegradable natural resource and the physical property and chemical reactivity of cellulose has attracted considerable research. Cellulose fibers exhibit many merits and advanced features such as the cheap price, abundant resource, light weight, biodegradability, and the capability for surface functionalization, which makes it a good matrix for incorporating various materials to construct composite with multiple advantages of cellulose fibers and guest materials. The β-cyclodextrins, polyacid, chitosan, and quinine were modified on the cellulose fibers to prepared cellulose fibers with special functionality [7,8,9,10].

With the development of nanotechnology, nanomaterials were incorporated with cellulose fibers and used in catalyst, supercapacitor electrodes, removal of metal ions, and biosensing [11,12,13,14]. Wang et al. deposited various TiO_2_ nanobelts on the surface of cellulose fibers to construct a functional composite, which show excellent photocatalytic activity in degrading methylene blue and antibacterial ability to *E. col* [15]. Liu and coworkers decorated cellulose fabric with reduced graphene to fabricate multi-functional fabrics, the cellulose fabric/graphene was successfully used in pressure sensing and energy harvesting [16]. Compared to molecules, metal oxide, and graphene, plasmonic nanoparticles (NP) show excellent features in optical and thermal aspects due to their plasmonic properties. When the plasmonic nanoparticles are irradiated by light, the free electrons on the surface of NPs are driven by the electric field to collectively oscillate at a resonant frequency, the phenomenon is named the surface plasmon resonance (SPR) [17,18]. The plasmonic NPs have been numerously applied in biological microscopy, optical sensors, and catalyst. The plasmonic NPs such as Ag and Au were incorporated with cellulose fibers to enable new capabilities to the prepared composites. Tian et al. have fabricated plasmonic absorbent cotton by depositing Ag colloid on cotton fiber, the plasmonic absorbent cotton was successfully used for adsorption and detection of thiram from cucumber by Raman spectroscopy, and the limit of detection achieved 0.1 ppm [19]. Zheng et al. decorated cotton fabrics with Au nanorods. Au nanorods enable cotton fiber to present a broad range of colors varying from brownish red through green to purplish red, which is assigned to the SPR feature of Au nanorods [20].

Surface-enhanced Raman scattering (SERS) spectroscopy is an advanced spectral technology as the sensitivity and selectivity. Since the discovery in the 1970s by Fleischmann [21], SERS has attracted strong interests from many researchers. The performance of SERS is dependent on the enhanced substrate. The enhancement effect of SERS substrate is mainly attributed to the localized surface plasmon resonance (LSPR) of the plasmonic materials [22,23,24,25]. With the development of nanotechnology, various plasmonic nanostructures have been developed and used for SERS sensing [24,26,27]. In order to detect analytes from objectives with irregular surfaces, flexible SERS substrates have been proposed. The soft matrix, such as PDMS film, cotton fabrics, cotton gauze, and cotton fibers, were used for constructing flexible SERS substrates [28]. Qu and coworkers prepared plasmonic cotton swab by assembling plasmonic NPs on cotton swab, the flexible capability of such plasmonic substrate allows for contacting the surface of cucumber through the simple swabbing process [29]. Cai and calibrators developed a flexible SERS substrate by decorating Ag NPs on natural woven fabrics and the plasmonic cotton fabrics showed excellent sensitivity for detecting *p*-aminothiophenol (10^−7^ M) [30]. Cotton fiber is not enough to meet the rapidly growing demand for textile fibers. The regenerated cellulose fiber from a waste resource is an effective strategy to meet fiber consumption in the world, which also provides sustainable solution for the recycled resource and waste accumulation [31].

In this study, we decorated the recycled cellulose fibers with Au NPs. The fiber was recycled from cellulosic waste such as paper and cardboard. The regenerated cellulose fiber-Au composites are flexible, cheap, and effective SERS substrates. The regenerated cellulose fiber was firstly functionalized with an amino group to graft positive charges. After that, the Au NPs were self-assembled onto the surface of fiber. The distribution of Au NPs on the fibers were controlled by the assembling time. These plasmonic cellulose were used as SERS substrates to detect R6G at a concentration down to 1 × 10^−9^ M. The flexible SERS cellulose fiber are highly effective for capturing analytes from a target with an irregular surface. These plasmonic SERS cellulose fiber is cheap, eco-friendly, and disposable, which offers a good platform for SERS sensing.

## 2. Experiment

### 2.1. Materials

Sodium hydroxyl (NaOH), gold (III) chloride trihydrate (HAuCl_4_ 3H_2_O), (3-Aminopropyl) trimethoxysilane (APTMS), 4-Mercaptobenzoicacid (4-MBA), and trisodium citrate dehydrates (Na_3_C_6_H_5_O_7_) were obtained from Innochem Sci. & Tech. Co., Ltd. (Beijing, China). Dimetridazole (DMZ) was purchased from Aladdin (Shanghai, China). The regenerated cellulose fiber from waste resource was supplied by the Biorefinery Group in Aalto University.

### 2.2. Synthesis of Au Colloid

The Au NPs (40–50 nm) used in this study were prepared as the previous report with minor modification [32]. Briefly, 100 mL of HAuCl_4_ 3H_2_O (1 mM) aqueous solution was boiled to reflux. After that, 2.3 mL of Na_3_C_6_H_5_O_7_ solution (1%) were dropped into the boiling solution, and kept boiling for half an hour, then cooled down to room temperature.

### 2.3. Fabrication of Regenerated Cellulose Fiber-Au Composite

The regenerated cellulose fiber was firstly treated with NaOH, briefly, 10 mg of regenerated cellulose fibers were soaked in 0.1 M of aqueous solution of NaOH for 20 min. After that the fiber was washed thoroughly with water and ethanol and dried in oven, and then immersed the fiber in 40 mL of 1% ethanol solution of APTMS for 5 h. The amino group was modified on the surface of cellulose fiber for grafting positive charge. The citrate capped Au NPs exhibited negative charge. A total of 2 mg of regenerated cellulose fibers modified with APTMS was soaked into 4 mL of Au colloid at different times. After that, the composite was washed with water and used for further study.

### 2.4. Apparatus

The UV-vis absorption spectra of Au colloid were measured on UV2400 UV–Vis spectrophotometer (Sunny Hengping Instrument, Shanghai, China). The scanning electron microscope (SEM) images of regenerated cellulose fiber before and after Au NPs decorating were collected on a SU8010 field emission scanning electron microscope (Hitachi, Tokyo Japan). Fourier transform infrared (FTIR) spectrum of regenerated cellulose fiber-Au composites were acquired from Spectrum GX spectrometer (PerkinElmer, Wellesley, MA, USA). The SERS measurement were carried on the portable Raman spectrometer (BWS465 iRman; B&W Tek, Newark, NJ, USA), and the laser was 785 nm.

### 2.5. Detection of Dimetridazole Using the Regenerated Cellulose Fiber-Au

The powder (1 mg) of dimetridazole was dissolved in 1 mL of aqueous solution of HCl (30 mM) at an initial concentration of 1000 ppm. After that, the dimetridazole solutions at different concentrations were prepared by diluting the initial solution of dimetridazole, and the dry regenerated cellulose fiber-Au was dipped into a solution of dimetridazole with different concentrations. After 1 min, the SERS regenerated cellulose fiber were transferred for Raman measurements. In order to investigate the SERS performance of regenerated cellulose fiber-Au to detect illegal drugs, the dimetridazole was mixed with meat. The SERS regenerated cellulose fiber were dipping from the sample and the SERS measurement was carried using a portable Raman spectrometer.

## 3. Results and Discussion

### 3.1. Preparation of Regenerated Cellulose Fiber-Au NPs Composites

**Figure 1:**
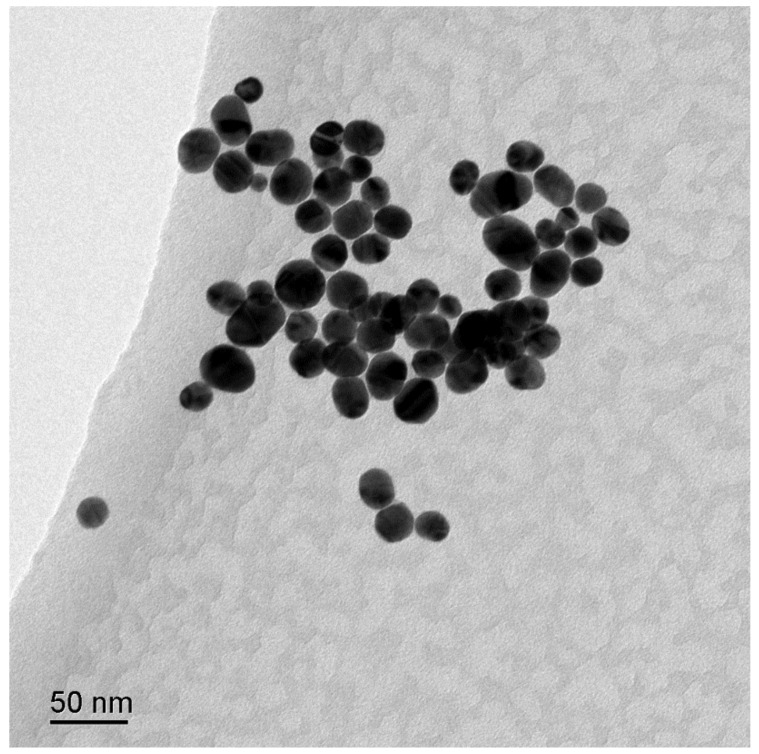
TEM image of colloidal gold nanoparticles.

The Au NPs used in this study were prepared through the trisodium citrate reduction method, in which the trisodium citrate functioned as a reducing and stabling reagent. The UV-vis spectra of Au colloid NPs were shown in Figure S1, in which the characteristic band at 526 nm is assigned to the localized surface plasmon resonance (LSPR) of Au NPs. The surface morphology of Au NPs was determined through the TEM image as shown in Figure 1. The spherical Au NPs with an average size of 40 nm were observed. The TEM image is consistent with UV-vis results. In order to observe the structure of Au NPs in detail, high-resolution transmission electron microscopy (HRTEM) observations were developed. The HRTEM image of single Au NP was presented in Figure S2, in which the lattice planes of the Au NP are observed. The 0.2355 nm interplanar distance is corresponding to the (111) planes of face centered cubic (fcc) of Au.

**Figure 2:**
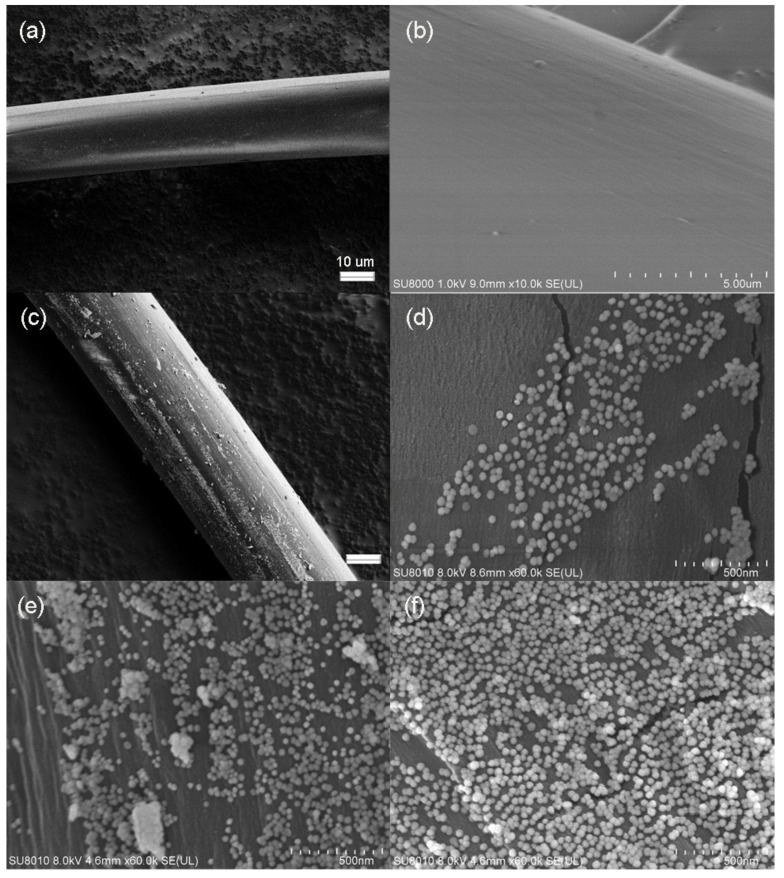
SEM images of regenerated cellulose fiber (**a**,**b**) and after decorating Au NPs (**c**–**f**).

The regenerated cellulose fiber showed a smooth surface as presented in the SEM image (Figure 2a,b). The diameter of the fiber was around 15 μm. After decorating Au NPs on the fiber, the rough surface fiber was observed as shown in Figure 2c. That result indicated that the Au NPs were assembled on the surface of the fiber successfully. SEM images with a high resolution were collected to observe the distribution of Au NPs on the surface of fiber. The different assemble time was used to control the density of Au NPs on the fiber during the self-assembly process. When the assembling time is 0.5 h, it could be observed that a small amount of Au NPs was decorated on the surface of the regenerated cellulose fiber (Figure 2d). As the assemble time increased to 2 h, the density of Au NPs distributed on the surface of cellulose fiber was increased significantly as present in Figure 2e. When the assembling time increases to 3 h, a dense layer of Au NPs was formed on the surface of fiber as shown in Figure 2f.

The property of the regenerated cellulose fiber before and after Au NPs decoration was also characterized by the FTIR spectrum as shown in Figure S3. The fiber composites were firstly cut to a small size and mixed with KBr for FTIR measurement. The feature IR peaks of cellulose were obtained. The peak at 3338 cm^−1^ is due to the stretching vibration of the OH group. The peaks at 2922 cm^−1^ and 2850 cm^−1^ were assigned to the anti-symmetrical vibration and symmetrical vibration of the methylene group. The prominent peak at 1060 cm^−1^ is attributed to the stretching vibration of the C–O–C group of pyranose ring in the cellulose [33]. The FTIR spectra of regenerated cellulose fiber after being modified with APTMS and Au NPs were similar with the blank fiber.

In order to investigate the decorating of Au NPs on fiber quantitatively, the UV-vis spectra were used to characterize the Au colloid after the self-assembling process. The intensities of LSPR bands of the Au colloid were decreased after removing the regenerated cellulose fibers from the colloid as shown in Figure S4. The decrease in the intensity of UV-vis spectra indicates that the amount of Au NPs remaining in colloid was reduced. The intensity of LSPR peak of Au colloid decreased as the assemble time increased, which indicated that more Au NPs decorated on fiber through the electrostatic interaction. The amount of Au NPs deposited on the regenerated cellulose fiber was estimated base on the variation of the UV-vis spectra. The initial concentration of Au colloid is nearly at 0.92 × 10^−10^ M according to the Lambert–Beer’s law, in which the molar extinction coefficient of colloid is 3.4 × 10^10^ M^−1^ cm^−1^ [34]. The 0.5-h assemble time corresponding to 1.48 × 10^−13^ M Au NPs decorated on the fiber. When the assembling time increased to 1 h, 2 h, 3 h, and 5 h, the amount of Au NPs on the cellulose fiber increased to 1.71 × 10^−13^ M, 2.31 × 10^−13^ M, 2.41 × 10^−13^ M, and 2.52 × 10^−13^ M, respectively.

**Figure 3:**
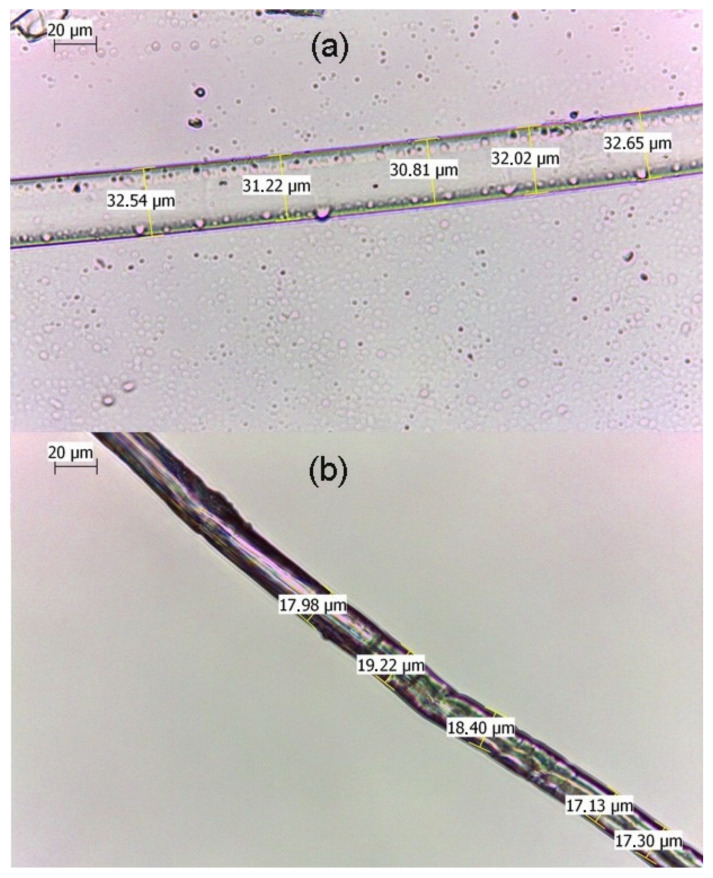
Microscope images of fiber-Au under wet (**a**) and dry (**b**) conditions.

The diameter of the fiber-Au composite was changed obviously under wet and dry conditions. As shown in Figure 3a, the diameter of the fiber-Au was nearly at 31 um under wet conditions. After dried in air conditions, the diameter of fiber-Au was decreased to 18 μm (Figure 3b). This variable feature can be explained as the cellulose of fiber were partially dissolved in the NaOH solutions, the free space in the fiber can adsorb more water during the swelling process. The shrinking process of the fiber-Au could decrease via swelling the distance of Au NPs, that could bring more “hot spots” for SERS measurement.

### 3.2. SERS Application of Regenerated Cellulose Fiber-Au Composites

**Figure 4:**
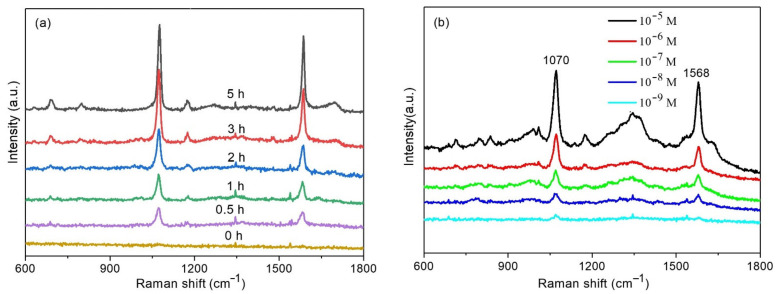
Raman spectra of MBA on cellulose fiber-Au with different assemble times (**a**), Raman spectra measured from the cellulose fiber-Au with a different concentration of MBA (**b**).

To evaluate the SERS performance of the plasmonic composite, MBA was chosen as the Raman probe molecule. Figure 4a presents the SERS spectra of MBA measured from different substrates. There are no Raman spectra of MBA observed from the regenerated cellulose fiber, which indicated no SERS enhancement effect on the regenerated cellulose fiber. After assembling Au NPs on the regenerated cellulose fiber, Raman spectra were obtained from the regenerated cellulose fiber-Au substrate. The SERS peaks mainly centered at 1070 and 1578 cm^−1^. The intense peak at 1070 cm^−1^ is attributed to the in-plane ring breathing vibration combined with a_1_ vibration mode of υ (C-S), the peak at 1578 cm^−1^ can be assigned to υ (CC), an a_1_ vibration mode [35]. The intensities of SERS spectra were increased as the increase of the assemble times, the reason is that more Au NPs decorated on the regenerated cellulose fiber. The dense Au NPs produce high electromagnetic fields and bring more ‘hot spots’ in the SERS measurement. A total of 3 h of assemble time was chosen for the SERS measurement to provide enough SERS enhancement. Figure 4b presented the Raman spectra of MBA at different concentrations ranging from 10^−5^ to 10^−9^ M. The intensities of Raman spectra decreased as the concentration of MBA decreased. The SERS spectra of MBA was still observed as the concentration went down to 10^−9^ M, which suggested the regenerated cellulose fiber-Au has effective SERS enhancement.

**Figure 5:**
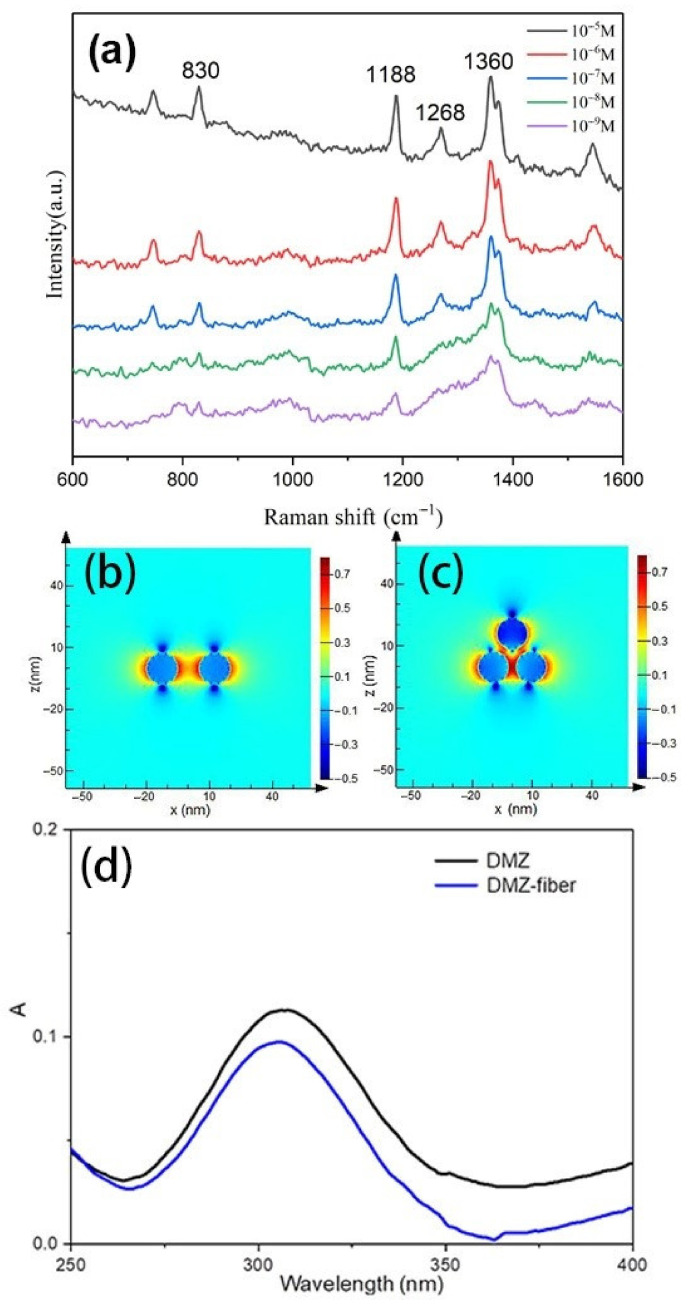
Raman spectra of DMZ with different concentrations: (**a**) FDTD simulations of SERS hot spots at Au NPs, dimer with a 10-nm gap (**b**) and trimer with a 4-nm gap (**c**), UV-vis spectra of DMZ (10^−5^ M), and after adsorbed by regenerated cellulose fiber-Au (**d**).

Dimetridazole (DMZ) is an effective veterinary drug that is usually used for controlling or treating bacterial and protozoal infections of animals. It has been proven that DMZ brings genotoxic and carcinogenic problems to public health. The European Union, United States, and China have forbidden the application of DMZ in food producing animals. The regenerated cellulose fiber-Au was used as the SERS substrate for detecting the DMZ molecule. The SERS spectra of DMZ with different concentrations were shown in Figure 5a. The intense Raman bands at 830, 1188, 1268, and 1360 cm^−1^ were presented. The prominent peak at 1188 cm^−1^ is due to the bending vibration of H–C–N, the most intense peak at 1360 cm^−1^ belongs to the deformational mode of H–C–N. The peak at 830 cm^−1^ results from the ring deformational mode combined with a wagging vibration of the NO_2_ group, the peak at 1268 cm^−1^ is attributed to the ring deformational mode. The peak at 1188 cm^−1^ can be observed even as the concentration of DMZ is down to 10^−9^ M. The enhanced effect of the plasmonic composite is comparable to the current existing flexible SERS substrate [36]. The excellent SERS performance could be attributed to two aspects. First, the dense Au NPs on the cellulose fiber could provide more ‘hot spots’ for SERS. A FDTD theoretical calculation was used to verify the intensity of the electric field of Au NPs, the distance between Au NPs on the regenerated cellulose fiber were set as 10 nm and 4 nm corresponding to dimer and trimer as shown in Figure 5b,c. The electric field within the gaps of trimer is higher than that from dimer, due to the coupled LSPR at a 4 nm gap distance, which provides a higher electric field than a 10-nm gap. Second, the cellulose fiber has an absorption capability [19], which enable it to adsorb DMZ from the solution. The UV-Vis spectra were used to verify the absorption capability of the composite as presented in Figure 5d. The absorption peak of DMZ was decreased obviously after the regenerated cellulose fiber-Au immersed in the solution of DMZ. More DMZ molecules were captured on the plasmonic composite during the process of adsorption, which benefited the SERS measurement as the sample enrichment effect.

**Figure 6:**
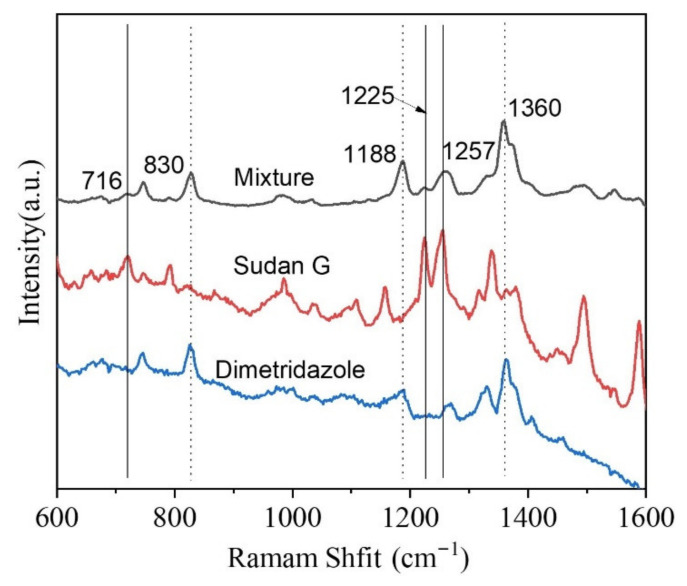
Raman spectra of DMZ, Sudan G, and their mixture from the regenerated cellulose fiber-Au.

In real life, there is usually more than one kind of molecule that exists in the target system and multiple components in the mixture may bring interference for analysis. Sudan G is a kind of industrial dye with azo group. As it can provide a bright red color, Sudan G has been used as an illegal additive in food. The illegal additive (Sudan G) and drug (DMZ) were mixed and used as analytes. The regenerated cellulose fiber-Au was dipped into the solution of mixture and transferred for SERS measurement. The SERS spectra from regenerated cellulose fiber-Au was presented in Figure 6. The characteristic Raman peaks attributed to Sudan G and DMZ were observed simultaneously. The detection of DMZ from the target with multiple components achieved by the substrate proposed in this study.

**Figure 7:**
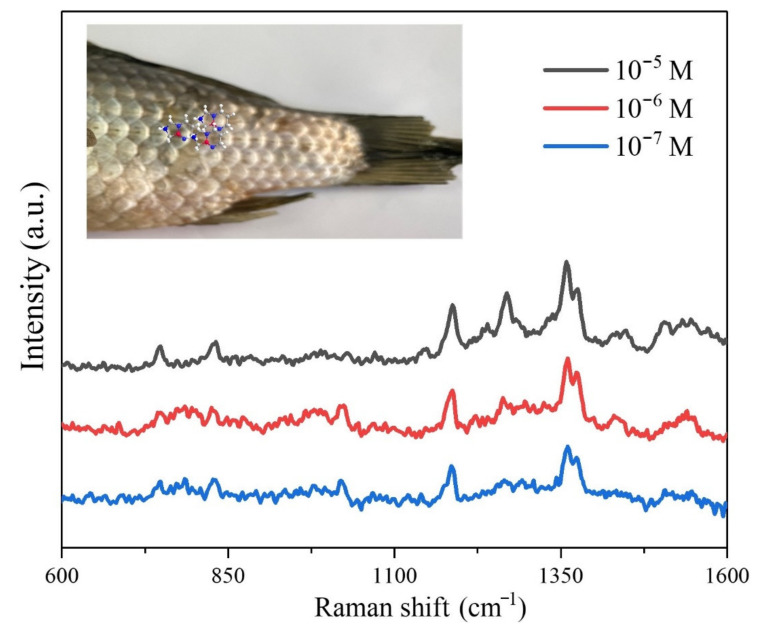
Swabbing detection of the DMZ from the surface of fish using fiber-Au as flexible SERS substrate.

In order to evaluate the SERS performance of fiber-Au in real food sample sensing, the fiber-Au composite was used as a flexible SERS substrate for DMZ detecting from fish. A total of 10 µL of DMZ with different concentrations were sprayed onto the surface of fish. The prepared fiber-Au was directly swabbed from the surface of a fish and for SERS spectra collecting. The SERS spectra are shown in Figure 7, the intensities of the SERS signal were decreased as the reduction of DMZ concentrations. The DMZ on the surface of fish could be easily adsorbed by fiber during the swabbing process.

## 4. Conclusions

The plasmonic composite was prepared based on regenerated cellulose fiber from waste paper. The regenerated cellulose fiber was firstly cationized with APTMS for grafting positive charges. The Au NPs were decorated on the surface of regenerated cellulose fiber by being deposited onto the surface of cotton gauze through electrostatic interaction. The distribution of Au NPs on the fiber could be controlled by adjusting the assembling time. The regenerated cellulose fiber-Au composite showed several advantages, such as eco-friendly, flexibility, adsorption ability, and active SERS enhancement. Proof-of-concept for the sensing application of the plasmonic composite was developed by identifying MBA and DMZ and the sensitivity could achieve 10^−9^ M. The fiber-Au also presented good selectivity for DMZ by SERS sensing. The plasmonic cellulose composite could be easily incorporated in paper or packages for in-situ monitoring of harmful ingredients in food and water.

## Data Availability

The data presented in this study are available on request from the corresponding author.

## Supplementary Materials

The following are available online at https://www.mdpi.com/article/10.3390/polym13132142/s1. Figure S1: UV-Vis spectra of colloidal gold nanoparticles, Figure S2: HRTEM image of Au NP, Figure S3: FTIR spectra of regenerated cellulose fiber and after modification, Figure S4: The UV-Vis spectra of Au colloid after exposure cellulose fibers with different assemble times.
