# Supplementary material for: Fabrication and Application of SERS-Active Cellulose Fibers Regenerated from Waste Resource
Source: Polymers (Basel). 2021 Jun 29;13(13):2142. doi: 10.3390/polym13132142 (PMC8272151; doi:10.3390/polym13132142)
Supplement: Supplementary file 1 [file polymers-13-02142-s001.zip › polymers-1288044-supplementary.pdf]

Fabrication and application of SERS-active cellulose fibers regenerated from waste resource

Shengjun Wang[a], Jiaqi Guo [b], Alan X. Wang[c] Yibo Ma[d], Xianming Kong[a*], Qian Yu[a*]

[a] School of Petrochemical Engineering, Liaoning Petrochemical University, Fushun, Liaoning 113001, P. R. China

[b] Jiangsu Co-Innovation Center for Efficient Processing and Utilization of Forest Resources and Joint International Research Lab of Lignocellulosic Functional Materials, Nanjing Forestry University, Nanjing 210037, P. R. China

[c] School of Electrical Engineering and Computer Science, Oregon State University, Corvallis, OR 97331, USA

[d] Department of Bioproducts and Biosystems, School of Chemical Engineering, Aalto University, FI-00076 Aalto, Finland

*Corresponding author: xmkong@lnpu.edu; qyu@lnpu.edu.cn

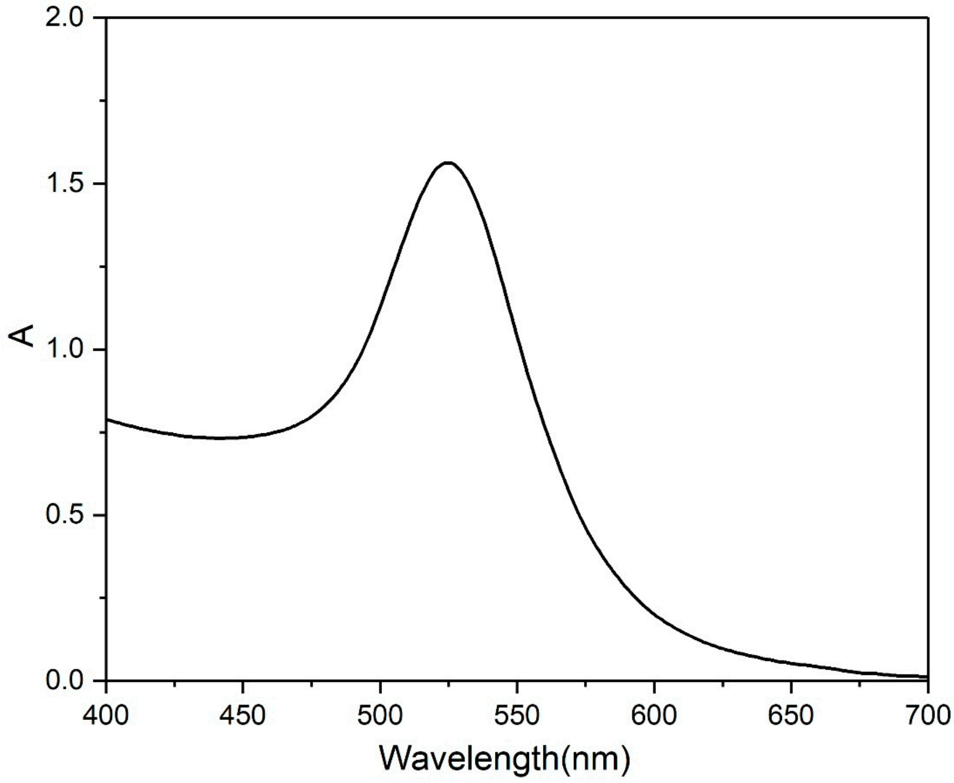

Figure S1. UV-Vis spectra of colloidal gold nanoparticles

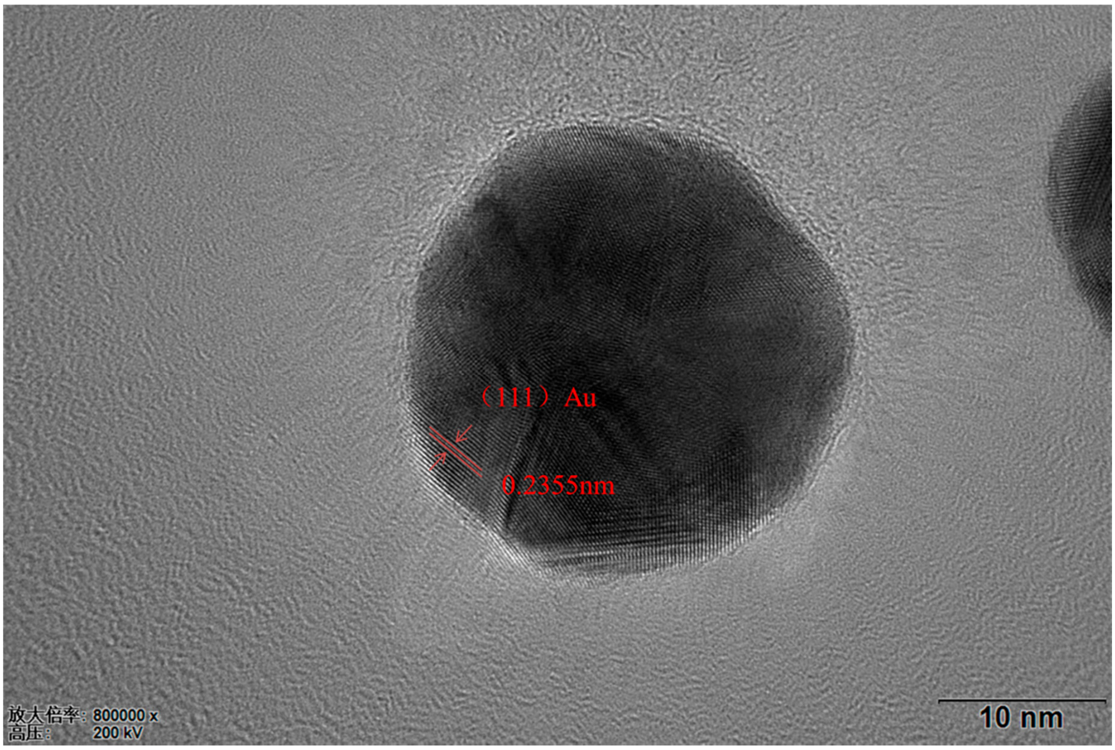

Figure S2. HRTEM image of Au NP

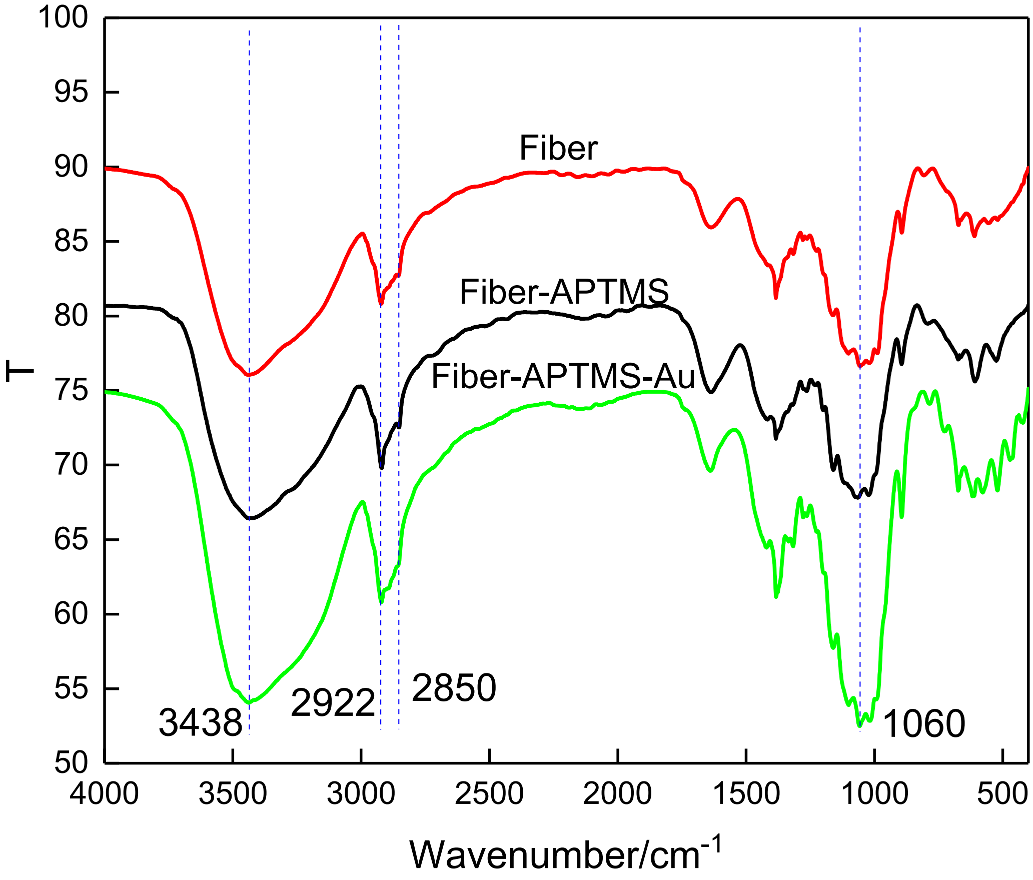

Figure S3. FTIR spectra of regenerated cellulose fiber and after modification

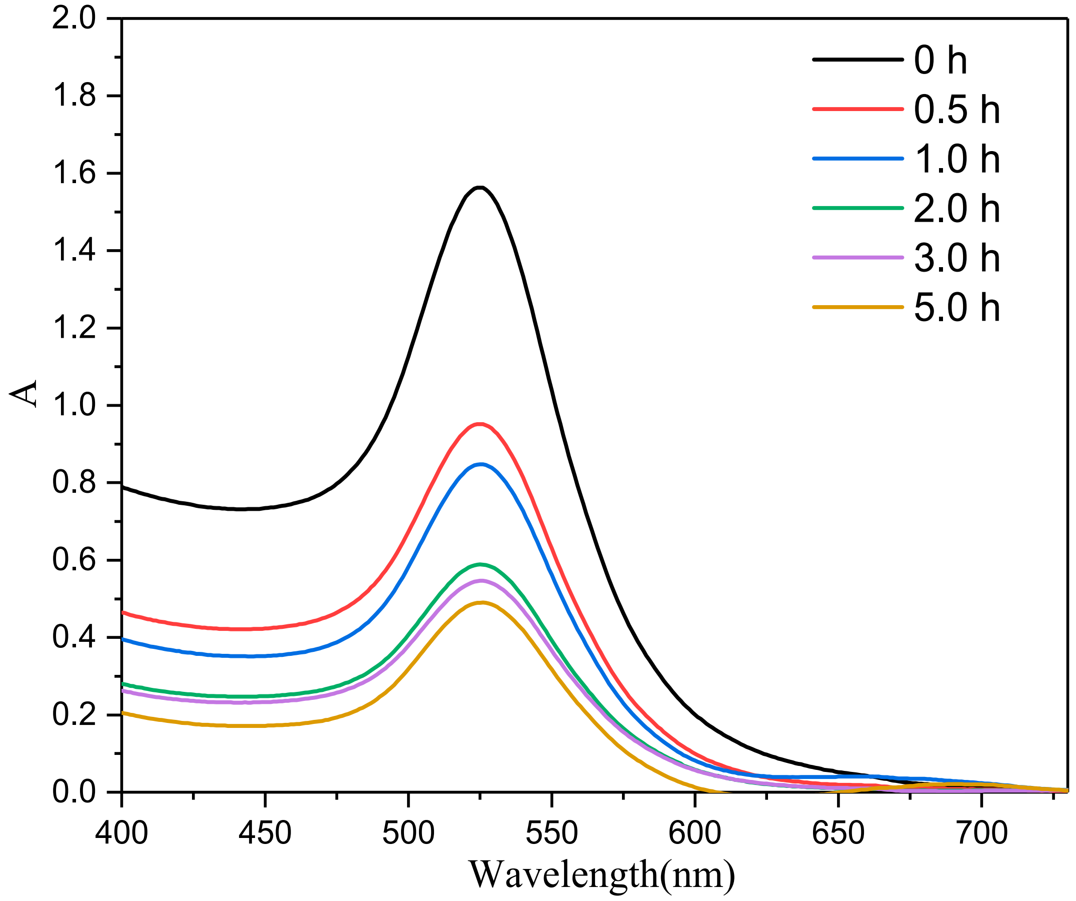

Figure S4. The UV-vis spectra of Au colloid after exposure cellulose fibers with different assemble times.
